# Supplementary material for: Robust deep learning-based gait event detection across various pathologies
Source: PLoS One. 2023 Aug 11;18(8):e0288555. doi: 10.1371/journal.pone.0288555 (PMC10420363; doi:10.1371/journal.pone.0288555)
Supplement: S1 Appendix — (ZIP) [file pone.0288555.s001.zip › supporting information.pdf]

Robust deep learning-based gait event detection across various pathologies - Supporting Information

Bernhard Dumphart[1,2,6*], Djordje Slijepcevic[3], Matthias Zeppelzauer[3], Andreas Kranzl[4], Fabian Unglaube[4], Arnold Baca[5], Brian Horsak[1,2]

1 Center for Digital Health & Social Innovation, St. Pölten University of Applied Sciences, Austria
2 Institute of Health Sciences, St. Pölten University of Applied Sciences, Austria
3 Institute of Creative\Media/Technologies, St. Pölten University of Applied Sciences, Austria
4 Laboratory of Gait and Movement Analysis, Orthopaedic Hospital Vienna-Speising, Austria
5 Centre for Sport Science and University Sports, University of Vienna, Austria
6 Doctoral School of Pharmaceutical, Nutritional and Sport Sciences, University of Vienna, Austria

* bernhard.dumphart@fhstp.ac.at

S1 Appendix: Supporting Information

The influence of the random initialization on the underlying models was tested and reported in the following tables. Table 1 to Table 4 report the results for the initial contact and foot off detection for our *IntellEvent* approach, separately. In Table 1 and Table 3 the different results for each random initialization (random seed) for all pathologies in milliseconds to the ground truth (force plate events) are visiualized for the initial contact and foot off. Each subsequent table (Table 2 and Table 4) show the descriptive results for the beforehand mentioned tables. The same results are visualized in Table5 to Table8 for the random initialization of the DeepEvent-retrained model for initial contact and foot off in the same pattern as described above.

In Table 9 and Table 10 results of the pair-wise Wilcoxon signed rank tests between the *IntellEvent*, DeepEvent-retrained, DeepEvent-original, and the heuristic approach are available for initial contact and foot off events for all pathologies. Furthermore, the effect size r is reported and significant values are bold.

Table 1. Results of the ten times random seed initialization of the *IntellEvent* initial contact model for all pathologies in milliseconds.

Random Seed	Malrotation Deformities	Club Foot	Drop Foot	ICP	Healthy Controls
**861**	2.69	3.89	6.55	5.16	2.46
**174**	2.97	3.89	5.94	5.26	3.46
**302**	3.13	3.73	5.37	5.04	2.81
**29**	2.71	4.01	5.98	5.14	2.77
**227**	2.68	3.89	5.94	5.18	2.67
**33**	2.79	3.32	6.22	5.20	3.31
**21**	2.67	3.45	5.41	4.93	2.50
**970**	2.85	4.11	6.13	6.17	2.59
**804**	2.90	3.75	6.41	5.34	2.54
**863**	2.89	3.85	5.41	5.17	2.81

Table 2. Descriptive Results concerning the ten times random initialization of the *IntellEvent* initial contact model for all pathologies in milliseconds.

	Malrotation Deformities	Club Foot	Drop Foot	ICP	Healthy Controls
**Min**	2.67	3.32	5.37	4.93	2.46
**Max**	3.13	4.11	6.55	6.17	3.46
**Range**	0.45	0.79	1.19	1.24	0.99
**Average**	2.83	3.79	5.94	5.26	2.79
**Median**	2.82	3.87	5.96	5.18	2.72
**Std.**	0.14	0.23	0.40	0.32	0.32

Table 3. Results of the ten times random seed initialization of the *IntellEvent* foot off model for all pathologies in milliseconds.

Random Seed	Malrotation Deformities	Club Foot	Drop Foot	ICP	Healthy Controls
**861**	7.91	9.36	12.63	11.72	8.90
**174**	8.83	9.55	10.21	11.98	9.66
**302**	8.42	8.75	10.26	11.52	9.43
**29**	7.84	9.90	10.83	11.37	8.26
**227**	7.87	8.69	9.88	11.28	8.34
**33**	8.10	9.25	11.11	11.67	8.71
**21**	7.72	9.78	9.73	11.35	8.23
**970**	7.61	9.31	12.82	12.38	8.36
**804**	7.95	9.35	11.21	11.38	8.13
**863**	9.56	9.19	12.06	12.68	10.63

Table 4. Descriptive results concerning the ten times random initialization of the *IntellEvent* foot off model for all pathologies in milliseconds.

	Malrotation Deformities	Club Foot	Drop Foot	ICP	Healthy Controls
**Min**	7.61	8.69	9.73	11.28	8.13
**Max**	9.56	9.90	12.82	12.68	10.63
**Range**	1.95	1.21	3.09	1.40	2.50
**Average**	8.18	9.31	11.07	11.73	8.87
**Median**	7.93	9.33	10.97	11.59	8.53
**Std.**	0.57	0.37	1.06	0.45	0.77

Table 5. Results of the ten times random seed initialization of the DeepEvent-retrained model for all pathologies in milliseconds for the initial contact events.

Random Seed	Malrotation Deformities	Club Foot	Drop Foot	ICP	Healthy Controls
**861**	3.74	4.61	6.46	6.90	4.10
**174**	3.32	4.29	6.55	6.75	3.50
**302**	3.50	3.97	6.93	6.37	4.06
**29**	3.38	4.09	7.31	6.79	3.85
**227**	3.71	4.55	6.79	6.39	4.18
**33**	3.24	3.83	7.03	6.21	3.46
**21**	3.23	3.89	5.94	5.87	3.93
**970**	3.35	4.35	7.50	6.62	3.68
**804**	3.14	3.93	6.65	5.85	3.74
**863**	3.28	3.95	7.12	6.37	3.72

Table 6. Descriptive results concerning the ten times random initialization of the DeepEvent-retrained model for all pathologies in milliseconds for the initial contact events.

	Malrotation Deformities	Club Foot	Drop Foot	ICP	Healthy Controls
**Min**	3.14	3.83	5.94	5.85	3.46
**Max**	3.74	4.61	7.50	6.90	4.18
**Range**	0.60	0.78	1.57	1.05	0.72
**Average**	3.39	4.15	6.83	6.41	3.82
**Median**	3.34	4.03	6.86	6.38	3.80
**Std.**	0.19	0.27	0.43	0.35	0.23

Table 7. Results of the ten times random seed initialization of the DeepEvent-retrained model for all pathologies in milliseconds for the foot off events.

Random Seed	Malrotation Deformities	Club Foot	Drop Foot	ICP	Healthy Controls
**861**	9.13	10.33	10.54	11.44	8.73
**174**	9.17	8.51	9.69	11.63	9.29
**302**	8.85	9.73	10.87	10.82	8.94
**29**	9.13	9.93	9.45	12.03	8.88
**227**	9.30	9.59	10.64	11.59	9.10
**33**	9.17	10.39	11.68	12.81	8.86
**21**	9.13	9.15	10.83	11.87	9.50
**970**	9.02	9.27	10.02	11.39	8.94
**804**	9.04	9.31	10.11	11.88	8.92
**863**	9.08	8.53	10.83	12.12	9.66

Table 8. Descriptive results concerning the ten times random initialization of the DeepEvent-retrained model for all pathologies in milliseconds for the foot off events.

	Malrotation Deformities	Club Foot	Drop Foot	ICP	Healthy Controls
**Min**	8.85	8.51	9.45	10.82	8.73
**Max**	9.30	10.39	11.68	12.81	9.66
**Range**	0.45	1.89	2.23	1.99	0.93
**Average**	9.10	9.47	10.47	11.76	9.08
**Median**	9.13	9.45	10.59	11.75	8.94
**Std.**	0.11	0.62	0.62	0.50	0.29

Table 9. Results of the Wicoxon signed rank tests between the initial contact events of the *IntellEvent*, DeepEvent-retrained, DeepEvent-original and the Ghoussayni et al. approach for all pathologies. All p-values are Bonferroni corrected by a value of three to mitigate alpha inflation. The effect size r was calculated using the Pearson correlation coefficient. Significant results are displayed bold.

	**Malrotation Deformities**			**Club Foot**			**Drop Foot**			**ICP**			**Healthy Control**		
	**Z**	**p**	***r***	**Z**	**p**	***r***	**Z**	**p**	***r***	**Z**	**p**	***r***	**Z**	**p**	***r***
**DeepEvent-retrained - *IntellEvent***	-5.1	**.000**	0.11	-1.9	.169	0.11	-2.1	.105	0.18	-4.8	**.000**	0.07	-5.5	**.000**	0.31
**Ghoussayni - *IntellEvent***	-28.9	**.000**	0.61	-12.2	**.000**	0.67	-9.7	**.000**	0.83	-23.0	**.000**	0.31	-8.5	**.000**	0.60
**Ghoussayni - DeepEvent-retrained**	-26.5	**.000**	0.56	-11.6	**.000**	0.64	-9.7	**.000**	0.82	-23.0	**.000**	0.31	-8.5	**.000**	0.48
**DeepEvent-original - DeepEvent-retrained**	-37.4	**.000**	0.79	-13.0	**.000**	0.72	-7.3	**.000**	0.62	-22.1	**.000**	0.70	-13.5	**.000**	0.75

Table 10. Results of the Wicoxon signed rank test between the foot off events of the *IntellEvent*, DeepEvent-retrained, DeepEvent-original and the Ghoussayni et al. approach for all pathologies. All p-values are Bonferroni corrected by a value of three to mitigate alpha inflation. The effect size r was calculated using the Pearson correlation coefficient. Significant results are displayed bold.

	**Malrotation Deformities**			**Club Foot**			**Drop Foot**			**ICP**			**Healthy Control**		
	**Z**	**p**	***r***	**Z**	**p**	***r***	**Z**	**p**	***r***	**Z**	**p**	***r***	**Z**	**p**	***r***
**DeepEvent-retrained - *IntellEvent***	-7.5	**.000**	0.16	-1.5	.420	0.08	-0.3	2.330	0.02	-2.3	.070	0.01	-1.3	.578	0.07
**Ghoussayni - *IntellEvent***	-26.1	**.000**	0.55	-8.4	**.000**	0.46	-6.4	**.000**	0.54	-14.7	**.000**	0.20	-8.1	**.000**	0.54
**Ghoussayni - DeepEvent-retrained**	-22.8	**.000**	0.48	-8.0	**.000**	0.44	-6.4	**.000**	0.54	-14.7	**.000**	0.20	-8.1	**.000**	0.46
**DeepEvent-original - DeepEvent-retrained**	-12.0	**.000**	0.25	-1.7	.086	0.09	-3.2	**.001**	0.27	-7.0	**.000**	0.22	-9.5	**.000**	0.53
