# Supplementary material for: Robust deep learning-based gait event detection across various pathologies
Source: PLoS One. 2023 Aug 11;18(8):e0288555. doi: 10.1371/journal.pone.0288555 (PMC10420363; doi:10.1371/journal.pone.0288555)
Supplement: Attachment — Submitted filename: Response to Reviewers.pdf [file pone.0288555.s002.pdf]

RESPONSE TO REVIEWERS' COMMENTS

Journal: Plos One
Submission ID: PONE-D-22-35192
Title: Robust deep learning-based gait event detection across various pathologies

Dear Editor and Reviewers,

thank you very much for giving us the opportunity to revise our manuscript. We are very grateful for the feedback and comments. These helped us to clarify the remaining inconsistencies and ambiguities in our article and improved our work. Please find below the reviewers' comments and our point-by-point responses. All changes in the manuscript are highlighted in blue color.

We have put great effort in revising the manuscript and are certain that your comments have helped us to increase the quality of our work.

Yours sincerely,

Bernhard Dumphart

Comments to the Author

Reviewer #1:

Comment #1: Link to the repo does not work

This paper proposes a deep learning approach for gait analysis, and more specifically for the detection of events in gait signals acquired in the laboratory. The experiments are conducted on a large (inaccessible) clinical dataset and a link is provided to the codes used (however, this link does not seem to be working currently).

Answer: Thank you for the comment. We have reworked the link and now everything is available. (**https://github.com/fhstp/IntellEvent**)

Comment #2: The proposed approach is compared to two state-of-the-art methods and allows to evaluate the interest of deep learning approaches for this application. Moreover, it is shown that the learning transfer problem is an open issue for this application. The authors searched for the best architecture using a grid search method. It would have been interesting to have an idea of the performance of the studied architectures, and in particular to understand the impact of the architecture design on the performance. Is this a crucial point to obtain reliable performances in clinical routine?

Answer: Thank you for your suggestion. We have incorporated the general trends regarding the hyperparameters derived from the grid search in the Method section as bullet points (**lines 197 to 210**). These bullet points allow the reader to understand the impact of various hyperparameters examined in our work.
The use of a grid search and evaluating different hyperparameter settings over the examined

performance measures (mean absolute error, true positive, false positive, and false negative rates) allows the identification of the most accurate and robust model for the presented dataset.

<u>Comment #3:</u> Have the authors addressed the problem of uncertainty estimation in this context, to further quantify the possibility of introducing these learning approaches into clinical routine?

<u>Answer:</u> Thank you for this comment. Our model is a probabilistic classifier which is able to predict a probability distribution over a set of classes – in our case for no event, (left or right) initial contact, and (left or right) foot off event. Probability helps to understand and quantify the expected variance in performance of predictive models when applied to new data which gives us information about how certain (or uncertain) an event is detected by our method.

To assess the generalizability to unseen data, we employed a train-test validation scheme with an additional validation set. The optimal hyperparameters for our models were determined using a grid search on the validation set. In a following step, we performed a sensitivity analysis to address the uncertainty mentioned by the reviewer. To this end, we trained the optimal models ten times on the training data using different random seeds and evaluated these models on the test data. In the paper we report the standard deviation over the ten runs (min: 0.14 – 1.06 ms in **line 282**). Further details can be found in the Appendix as well (**line 284**). For visualization purposes we decided to report the values of the best model (reported for all other approaches as well)

<u>Comment #4:</u> And for example, what are the worst detections? Are they related to a particular walking profile? Are the errors similar?

<u>Answer:</u> Thank you for this question. We have added information about the false positive (FP) temporal errors (= worst detections) in the Results section. Please refer to **lines 313 to 320** in the manuscript.

Reviewer #2:

The authors have developed a new deep learning (DL)-based algorithm (IntellEvent) to robustly identify gait cycle events from an optical motion capture system using reflective markers. The authors have performed a very detailed analysis of the algorithm performance in detecting events (foot-off, FO, and initial contact, IC) across different pathological gait patterns and compared it to both a validated heuristics-based algorithm and a relevant DL-based algorithm (i.e., DeepEvent). Besides testing its validity, the authors also address the applicability of a DL-based algorithm on an out-of-distribution dataset by evaluating the performance of DeepEvent on a new gait dataset. Their newly proposed DL-algoritm (IntellEvent) achieved excellent results, and as such the authors have developed a sound and useful algorithm for events detection across different pathologies. To further improve the manuscript, the authors may consider addressing the following point that were raised:

Comment #1: Introduction Lines 49 – 52

The authors state that the modified version of Ghoussayni et al. achieved the most promising results overall for both the FO and IC detection. They may want to refer that other research (Ulrich et al., 2019, doi: 10.1016/j.jbiomech.2019.05.006, Hendershot et al., 2016, J. Biomech. 49, 4146-4149) has shown that a combination of (O'Connor et al., 2007, Gait & Posture) for IC detection and (Zeni et al.,

2008, Gait & Posture) for FO detection works best for gait event detection during treadmill walking and over ground turning. This actually strengthens the line of argumentation from the authors, that there is no consensus on which heuristics-based algorithm works best for event detection from marker trajectories.

Answer: Thank you for your suggestions to strengthen the line of argumentation. We have incorporated both references in the Introduction. Please refer to **lines 56 to 61** in the manuscript.

Comment #2: Methods Lines 149 – 152

The authors state that two separate models were trained, because otherwise problems occurred in detecting the first FO and the last IC in most trials. Could this be because the initial and terminating step are different from the "intermediate" or "steady-state" steps? In some gait research they leave out the initial and final two to three steps of analysis. Would this have been an option as well for the case that only one model is used?

Answer: Thank you for this input. We also have discussed in our team that this could be a problem but actually all participants should be in steady-state walking due to the setup of the gait laboratory in the Orthopaedic Hostpial Vienna Speising (four to five steps are made before and after the capture volume starts/ends).
The reason for using two separate models lies in a "bias" of the ground reaction force events where the first event in each trial is an IC and the last event is always a FO event. In our initial approach, the model learned this pattern but when we applied it on the entire gait trial (containing also steps before and after the ground reaction force plates) the first and last event may deviate from the ground truth pattern and therefore were often not detected. To overcome this problem, our approach utilizes two separate models, i.e., an IC detection model and an FO detection model. Since there are two separate models for the two events, each model can focus on the event for which it was trained, and the "bias" in the order of events in the ground reaction force data does not cause a bias in the trained models. A further advantage is that we can use different features (e.g., different markers) and different model architectures for the detection of the two different events.

Comment #3: Discussion Lines 427 – 430

The authors state that " … With respect to RQ2, we conclude that using available deep learning-based approaches, which have been trained on data from a specific laboratory, need to be applied to different gait laboratories with care because a certain bias could be introduced due to different sampling frequencies or setup differences between the laboratories." Does this mean that using IntellEvent on our gait dataset will also be prone to bias, in other words the use of IntellEvent is also limited to a certain marker and walking test setup?

Answer: Thank you for this question. To date we could evaluate the bias introduced by different gait laboratories only with the publicly available model proposed by Lempereur et al. (2020). To this end, we evaluated this model also on our data. However, due to restrictions in data availability (e.g., Lempereur et al. didn't published the data from their gait laboratory), we couldn't evaluate similar biases for the IntellEvent algorithm. However, we utilized several strategies to mitigate the source for this type of bias: i) the data that IntellEvent was trained on utilized markers (heel, toe, ankle) that should be available in almost all conventional clinical marker sets; ii) we trained our models only on steps for which we have the ground truth data from ground reaction force plates, thus our models should work independently of the laboratory size, and iii) for the walking test setup (for example: different laboratory sizes lead to different walking speeds) we have introduced normalization of the trajectories. This leads us to our future research, where we want to investigate a multi-center gait event detection algorithm to minimize all these biases as well as to publish a transfer learning-based

version of IntellEvent, where new features can be easily learned and adapted for the specific laboratory.
We have clarified in the manuscript that IntellEvent could be subject to these biases (see **lines 477 to 481**) to avoid misconceptions.

Reviewer 3:

Comment #1: In the paper the authors proposed a novel deep learning-based gait event detection algorithm called IntellEvent based on stacked bi-directional LSTM. However, I am unable to find any novelty in this work. In line number 66-67, the authors mentioned that "Kidzinski et al. [12] utilized a stacked bi-directional long short-term memory (LSTM) neural network architecture to detect gait cycle events in children with mostly neurological disorders". Then, how there proposed stacked bi-directional LSTM is a novel approach.

Answer: We want to thank the reviewer for critically questioning the contribution of our work. This has led us to two important conclusions: First, that we inaccurately described the state-of-the-art, especially with respect to Kidzinski et al. (2019) and second that the description of the contribution of our work was not to the point.

Considering the first issue, we have to point out that Kidzinski et al. (2019) did not used a bi-directional LSTM approach, but instead a classical LSTM. They report inferior performance compared to our approach. See for example the following comparison of temporal detection accuracy: IntellEvent IC: 5.4 ms vs. Kidzinski et al.: 10 ms and IntellEvent FO: 11.3 ms vs. Kidzinski et al.: 13 ms. These results originate even from the worst performing class in our experiments. For the other classes the margin is even higher: (IntellEvent FC: 2.7, 3.5, 5.4, 4.9, 2.5 ms; FO: 7.9, 8.7, 9.9, 11.3, 8.3 ms). We have clarified the difference between the proposed approach and the one by Kidzinski et al. in the manuscript and have highlighted the performance differences, see **lines 71 to 79**.

Considering the description of the contribution of the paper, we want to clarify that we do not see the presented network architecture as the main contribution, but rather the overall approach presented in the paper. More concretely, the main contributions of the paper include: (i) evaluating the performance of ML-based gait event detection methods on various pathologies using one of the most comprehensive datasets in the literature, (ii) a novel event detection approach that is based on two separate deep learning models for the two event types that are trained using only ground truth events from force plates as compared to state-of-the-art methods, which have used all available (e.g., manually set) events, and (iii) exploring the presence of bias in a publicly available state-of-the-art ML model utilizing data from our gait laboratory to understand current limitations of ML-based gait event detection methods.

We have clarified the contribution in the paper to avoid raising misleading expectations for the reader, see **lines 130 – 138**. Please note, that an additional contribution (added during this paper revision) are the general trends for each examined hyperparameter derived from our comprehensive grid search (initiated by Reviewer #2). We hope that above clarifications help to better assess the actual contribution of the paper and its embedding in the related work.

Comment #2: In line number 12-123, the authors mentioned "The source code for IntellEvent is available on GitHub (https://github.com/fhstp/IntellEvent) with detailed documentation for training, retraining, and integrating the algorithm in an existing motion capturing pipeline". However, the link is giving error 404 message.

Answer: Thank you for the comment. We have reworked the link and now everything is available. **(https://github.com/fhstp/IntellEvent)**

Comment #3: In line number 156-158, the authors mentioned "To find a suitable configuration for our architecture, hyperparameters of the stacked LSTM model were optimized by conducting a grid search including the number of layers, hidden units, dropout values, and sample weights". However, the authors did not mention the best hyperparameter values.

Answer: Thank you for this hint, we have updated the text and included all hyperparameters that were fine-tuned during the grid search (**lines 180 182**). Due to the input from Reviewer #2, we evaluated the general trends regarding the hyperparameters derived from the grid search. Please refer to **lines 197 to 210** in the manuscript.

Comment #4: Explanation of figure 1 is totally missing.

Answer: We can understand the confusion, but due to the Plos One guidelines the figures are uploaded separately as individual files and are therefore at the bottom of the submission. (https://journals.plos.org/plosone/s/file?id=wjVg/PLOSOne_formatting_sample_main_body.pdf). Figure captions are in the manuscript (**Fig 1: after line 231, Fig 2: after line 323, Fig 3: after line 328**) where the figures will appear and after the paragraph in which they are first mentioned.

Comment #5: How the proposed stacked bi-directional LSTM architecture is different from the architecture that was proposed in Kidzinski et al. [12].

Answer: Thank you for this indication. We have clarified the difference between the proposed approach and the one by Kidzinski et al. in the manuscript and have highlighted the performance differences, see **lines 71 to 79**. Please refer to **Comment #1** of your Review for a more detailed explanation.

Comment #6: The tables and figures should be in the proper position of the manuscript.
Answer: We can understand the confusion, but due to the Plos One guidelines the figures are uploaded separately as individual files and are therefore at the bottom of the submission. (https://journals.plos.org/plosone/s/file?id=wjVg/PLOSOne_formatting_sample_main_body.pdf). Figure captions are in the manuscript (**Fig 1: after line 231, Fig 2: after line 323, Fig 3: after line 328**) where the figures will appear and after the paragraph in which they are first mentioned as well as the tables.
